# Fused Raman spectroscopic analysis of blood and saliva delivers high accuracy for head and neck cancer diagnostics

**DOI:** 10.1038/s41598-022-22197-x

**Published:** 2022-11-02

**Authors:** Hanna J. Koster, Antonio Guillen-Perez, Juan Sebastian Gomez-Diaz, Maria Navas-Moreno, Andrew C. Birkeland, Randy P. Carney

**Affiliations:** 1 Biomedical Engineering, University of California, Davis, CA USA; 2 Electrical and Computer Engineering, University of California, Davis, CA USA; 3 illumifyDx, Inc., Broomfield, CO USA; 4 Department of Otolaryngology, University of California, CA Davis, USA

**Keywords:** Oral cancer detection, Raman spectroscopy, Diagnostic markers

## Abstract

As a rapid, label-free, non-destructive analytical measurement requiring little to no sample preparation, Raman spectroscopy shows great promise for liquid biopsy cancer detection and diagnosis. We carried out Raman analysis and mass spectrometry of plasma and saliva from more than 50 subjects in a cohort of head and neck cancer patients and benign controls (e.g., patients with benign oral masses). Unsupervised data models were built to assess diagnostic performance. Raman spectra collected from either biofluid provided moderate performance to discriminate cancer samples. However, by fusing together the Raman spectra of plasma and saliva for each patient, subsequent analytical models delivered an impressive sensitivity, specificity, and accuracy of 96.3%, 85.7%, and 91.7%, respectively. We further confirmed that the metabolites driving the differences in Raman spectra for our models are among the same ones that drive mass spectrometry models, unifying the two techniques and validating the underlying ability of Raman to assess metabolite composition. This study bolsters the relevance of Raman to provide additive value by probing the unique chemical compositions across biofluid sources. Ultimately, we show that a simple data augmentation routine of fusing plasma and saliva spectra provided significantly higher clinical value than either biofluid alone, pushing forward the potential of clinical translation of Raman spectroscopy for liquid biopsy cancer diagnostics.

## Introduction

When cancer is detected early, treatments are more effective and survival improves. Current diagnostic modalities of imaging (e.g., ultrasound, CT, MRI, PET) and solid biopsy with pathology and immunohistochemistry are either invasive, time-consuming, or frequently inaccurate, therefore not ideal for routine screening of at-risk patients^1^. There is a critical need to develop rapid, inexpensive, and accurate new platforms that identify tumor associated features in circulating biofluids in the earliest stages. Liquid biomarkers chemical analytes of interest present within a patient biofluid could provide significant clinical and economic benefits, paving the way towards precision medicine and patient-centered care^2^.

It is unlikely that a single biomarker will detect all types of cancer or reliably inform clinical care throughout diagnosis and treatment; therefore, techniques capable of analyzing signatures representing a broad range of molecules are needed. Omics platforms based on mass spectrometry (MS), encompassing genomics, transcriptomics, proteomics, lipidomics, and metabolomics, have transformed our understanding of cancer molecular biology^3–5^. Yet such approaches are relatively slow, high in cost and complexity, low throughput, and require large sample volumes, thus are impractical for many stages of clinical care^6–8^. These limitations are especially prohibitive for large scale routine cancer screening, thus there are huge advantages to moving towards diagnostic platforms that do not rely on MS.

Instead, an ideal diagnostic test would be rapid, real-time, reagent-free, non-destructive, inexpensive, highly accurate, and require minimal background training and minute amounts of minimally- or non-invasively collected sample (e.g., plasma, urine, or saliva). Raman spectroscopy (RS) addresses many of these needs: it requires little to no sample preparation, is non-destructive, does not need exogenous dyes or labelling agents, and can be performed directly in aqueous solutions^9^. RS has been applied to diagnose many cancers to date, including breast^10^, pancreatic^11^, skin^12^, colon^13^, gastric^14^, and lung cancer^15^. In head and neck cancer (HNC), prior applications mainly focused on tissue, either as a screening tool^16,17^, for identification of potential recurrence^18^, or general discrimination between normal and cancerous tissues^19–26^. But there is little work done using RS to identify and validate early-stage HNC liquid biomarkers, particularly comparing against benign disease or diagnostic staging. In addition, typical Raman research is carried out on smaller datasets which leads to over-fitting of data and misinterpretation^27^. When applied to larger cohorts, the sensitivity and specificity of such platforms typically drops due to increased complexity and inter-patient variation^28^. These limitations have hindered the clinical adoption of Raman-based platforms.

Of importance to note is that many studies related to cancer detection are carried out using surface-enhanced Raman scattering (SERS), a highly sensitive extension of Raman that uses nanoscale metallic features to induce a strong electromagnetic enhancement^29–33^. SERS has many of the same features mentioned above but measurements can be done more quickly with a stronger signal. However, SERS struggles with reproducibility due to inherent signal heterogeneity, making it difficult to validate the results obtained^34^. For this reason, we decided to utilize conventional Raman in this study.

RS is an attractive diagnostic tool, but there remain some obstacles that need to be addressed to maximize clinical translation potential. Typical Raman instruments tend to have a large physical footprint. Efforts in producing miniaturized, portable systems are advancing, but it is not clear that their resolution would permit the diagnostic performance achieved in this study. Another drawback currently is the lack of automation for fast and easy measurements. Currently, RS requires highly trained users for sample measurement and data analysis. Furthermore, an accounting for the range of preanalytical variables that may affect measurement was not carried out in this work. It is not clear how diagnostic model performance would be affected by sample storage conditions, time of day for liquid biopsy collection, or total volumes collected (to name a few). Future work to address these pitfalls could increase the translational potential of RS as an indispensable clinical tool.

As of 2018, head and neck cancer (HNC) was the seventh most prevalent cancer worldwide with 890,000 new cases and 450,000 deaths^35^. Although cases linked with tobacco and alcohol use have been on the decline, cases of human papillomavirus (HPV)-associated HNC cancer are increasing, mainly induced by HPV^36^. Approximately 30–40% of patients are diagnosed with stage I or II HNC, which is typically curable with surgery or radiotherapy alone and increases long-term survival rates to 70–90% for those individuals^37^. However, this leaves more than 60% of patients with HNC presenting in stage III or IV, which carries a high risk of distant metastasis, local recurrence, and a 5-year overall survival of 50%^38^. It is evident from these reported numbers that HNC diagnosis could benefit from finding and validating early-stage biomarkers that can be correlated with the disease progression and further monitored to assess patient reaction to treatment.

Another interesting angle of this research involves the augmentation of data, in this case by combining the Raman spectra of multiple biofluids. More diagnostic information may be uncovered by creating these stitched biofluid datasets, leading to a potential increase in diagnostic ability of our platform. This type of low-level data combination has been utilized before in the context of assessing pollutants in oils^39^, clay minerals^40^, or analyzing the purity of red meats to uncover food fraud^41,42^. Some initial work applying Raman spectra concatenation has been applied to biological samples, but this work was done by combining different types of data (in this case Raman and MALDI spectrometric imaging)^43^. We believe this is the first time Raman data augmentation has been performed using multiple biofluid sources rather than analytical techniques.

In this study we carried out RS measurements of paired blood and saliva for a 53-person cohort. Using chemical standards of metabolites identified by MS on a subset of those patients, we confirmed that the spectral features upon which discrimination is based in RS are associated with the same biomolecules identified by MS. We determined optimal pre-analytical variables (e.g., native vs. dried biofluid) that maximized model performance. Our major finding is that accuracy, sensitivity, and specificity approaching, and even surpassing MS, could be achieved by RS using an innovative approach to stitch together spectra from plasma and saliva for each patient. To the best of our knowledge, this is the first study that uses RS to directly validate metabolites from patient biofluids, as well as to analyze combination biofluid spectra to achieve highly accurate diagnostic performance.

## Methods

### Clinical biofluid collection and processing

Patients were consented to blood draws and non-stimulated saliva collection during scheduled head and neck cancer surgeries in the University of California, Davis Department of Otolaryngology and all methods were performed in accordance with the relevant guidelines and regulations. A cohort of 53 patients, comprised of 34 cancer and 19 benign control patients, were collected. Sample collection was limited samples to patients specifically diagnosed with squamous cell carcinoma (SCC), which represents over 90% of all HNC cases^44^. As the control group for this study, we utilized samples from individuals with scheduled head and neck surgeries for benign conditions (e.g., tonsillectomies or benign mass removal). We further segmented our cancer patient cohort into early (stage I, II) and late (stage III, IV) to investigate the potential of RS for diagnostic staging. Details about the individual patients (e.g., diagnosis, staging, gender, age) can be found in Table S1. Saliva was aliquoted into Eppendorf tubes with a volume ranging from 200 to 1000 μL and frozen at − 80 °C until retrieval. Blood draws were isolated to plasma and aliquoted into four tubes each with volumes ranging from 200 to 500 μL and frozen at − 80 °C until retrieval. Saliva was thawed in an ice bath and further aliquoted into smaller storage volumes if the original sample volume allowed. One tube from each patient’s plasma and saliva were separated and sent to the West Coast Metabolomics Center for GC-MS analysis. No further isolation was performed before Raman measurements were done.

### Gas chromatography and mass spectrometry analysis

Metabolomics data was collected for primary and polar metabolites using gas chromatography-time of flight mass spectrometry (GC-TOF MS) on a Leco Pegasus IV time of flight mass spectrometer with Agilent 7890A gas chromatograph and 7693 autosampler^45^. Briefly, 30 μL of either plasma or saliva was extracted at − 20 °C with 1 mL degassed isopropanol/acetonitrile/water (3/3/2). Extracts were dried down, cleaned from triacylglycerides using acetonitrile/water (1/1), and derivatized with methoxyamine and trimethylsilylation. Samples (0.5 μL) were injected at 250 °C to a 30 m rtx5-SilMS column, ramped from 50 to 300 °C at 15 °C/min, and analyzed by − 70 eV electron ionization at 17 spectra/s. Raw data were deconvoluted and processed using ChromaTOF vs. 4.1 and uploaded to the UC Davis BinBase database^46^ for data curation and compound identification^47^. Resultant data were normalized by to their respective average MTIC for each sample type.

### Raman analysis

Plasma and saliva were analyzed in both a native and dried state. Biofluid samples were thawed on ice and 2 μL was pipetted onto a quartz coverslip. For native measurements, the quartz coverslip was immediately placed on our Raman microscope and spectra were collected with the laser focused through the coverslip and 2 μm into the sample. For dried measurements, the native spots were allowed to dry for 15 min under ambient conditions prior to measurement and the laser was focused slightly above the interface of the sample and coverslip. For the dried experiments, the biofluids dried in concentrated spots that appeared homogeneous with no apparent coffee rings. However, to account for potential drying effects, spectra were taken from 5 areas distributed throughout the dried spot, including near the edges and around the center area. For analytical metabolite standards, powder or liquid for each was placed directly from the chemical container onto a clean coverslip with no modifications and the laser was focused 2 μm into the sample. Spectra were acquired using a custom-built inverted Raman scanning confocal microscope with excitation wavelength of 785 nm and a 60x, 1.2 NA water immersion objective on an inverted IX73 Olympus microscope. An Andor Kymera-3281-C spectrophotometer and Newton DU920P-BR-DD CCD camera were used for Raman spectra capture and Solis v4.31.30005.0 software was used for initial processing. For measurements, exposure time was 30 sec per scan with laser power of 65 mW. Substrates were randomly sampled at 5 regions and averaged.

### Data analysis

Partial least-squares discriminant analysis (PLS-DA), a supervised multivariate dimensionality-reduction tool^48^, was used to analyze the large omics datasets. Samples were labeled (in this case as either cancer or benign control) and projected onto a new space as observable variables for modeling the best linear regression to split the pre-identified groups^48^. Single cross validation was used, where some individuals were set aside to create a validation set, and the remaining individuals used to develop classification models. This procedure is repeated with a new set of individuals reserved until all individuals have been used only once in a validation set. The model with the lowest total prediction error was selected as the best. Analysis of the spectral data was performed using MATLAB v2021a (MathWorks, MA, USA) via a custom script. Cropping, penalized least-squares (PLS) background correction^49^, smoothing^50^, and unit normalization were applied for spectral pre-processing. Spectra containing cosmic rays were removed from analysis. Spectra were subjected to PCA and LDA/QDA based on the corresponding MATLAB built-in functions applied to a custom script.

### Biofluid data stitching

For each of the patients with both biofluid samples present, the average plasma spectra and average saliva spectra intensity values were exported into a new .txt file. Arbitrary values were assigned to the x-axis starting with 1 and ending with 1694. New files (with plasma first, followed by saliva) were analyzed with the same processing parameters outlined above.

### Live subject statement

Patients were consented to blood draws and saliva collection during scheduled head and neck cancer surgeries in the University of California, Davis Department of Otolaryngology. All experiments were performed in accordance with an approved protocol by the UC Davis Institutional Review Board committee (IRB #: 930499-8). Informed consent was obtained for all patients.

## Results

### Mass spectrometry analysis

**Figure 1:**
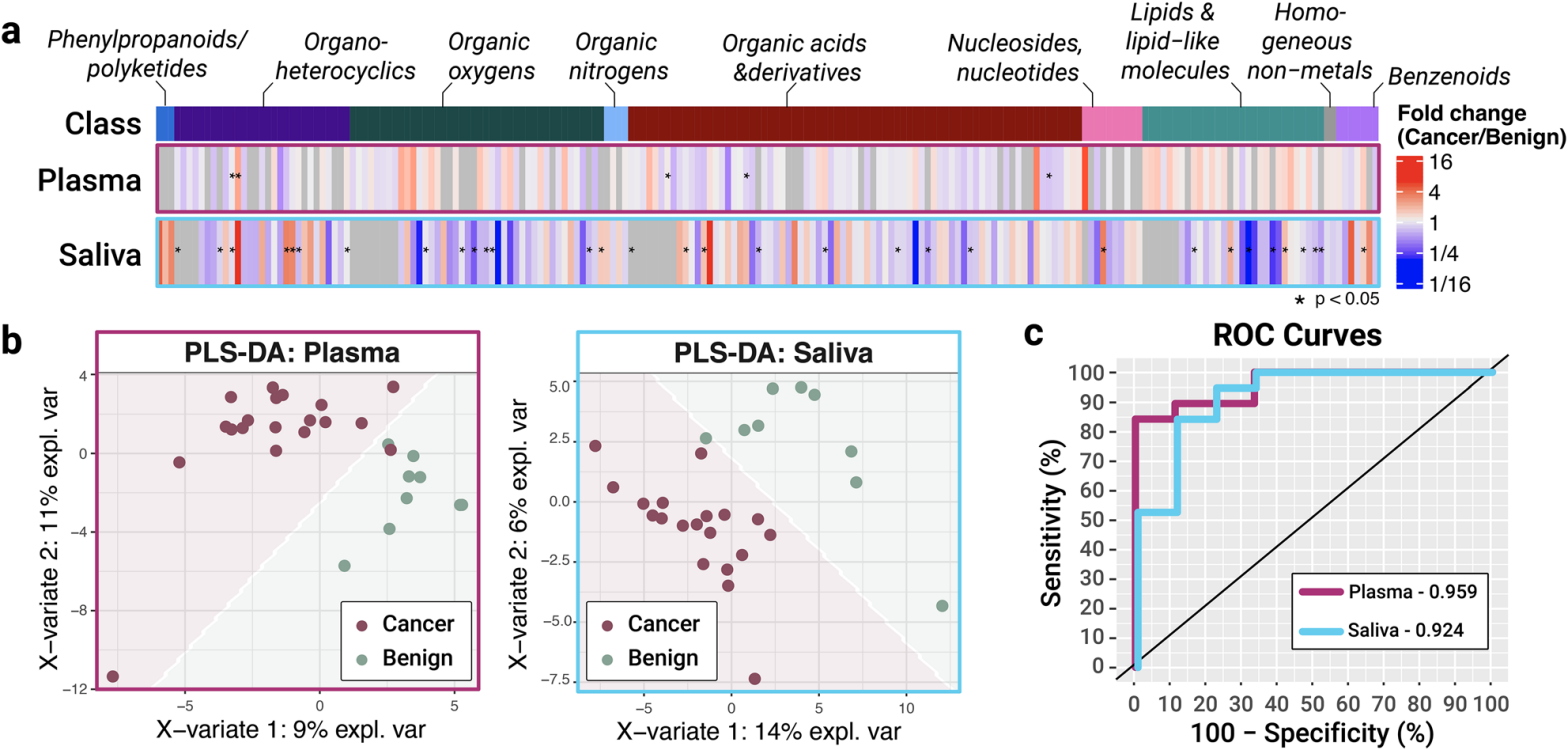
GS-TOF-MS analysis of plasma and saliva collected from SSC vs benign control cases. (**a**) Metabolites grouped by chemical class were identified from either plasma (top) or saliva (bottom). Color scale represents fold change for cancer/benign, with red representing a higher prevalence in cancer biofluids and blue a higher prevalence in the controls. (**b**) PLS-DA of the pre-labeled cancer and control samples for plasma and saliva reveals excellent discrimination with only a single sample misclassified in each biofluid. (**c**) ROC curves enable calculation of AUC to be 95.9% and 92.4% for plasma and saliva, respectively.

For the initial subset of 28 patients (19 SSC and 9 control cases) collected for this study, plasma and saliva biofluids for each patient were measured by gas chromatography - time of flight mass spectrometry (GC-TOF-MS) for primary and polar metabolite identification^45^. Metabolites grouped by structural class were ranked based on their fold-change in SSC vs. control samples (Fig. 1a). The highest fold changes occur within distinct classes and differ widely between the two biofluid types. Saliva yielded more polarized data compared to plasma, with a higher number of metabolites featuring a significant fold change between cancer vs. benign controls. Nevertheless, both plasma and saliva each provided high diagnostic value when subjected to partial least-squares discriminant analysis (PLS-DA). The first two X-variates in the model yielded the best model performance. For plasma or saliva, this represented 20% of the total variance in each case. For each biofluid, only one sample was misclassified (Fig. 1b). Receiver operating characteristic (ROC) curves were generated by plotting the true positive rate against the false positive rate for plasma and saliva (Fig. 1c). The area under curve (AUC) values were 95.9% for plasma and 92.4% for saliva. This was the highest reported AUC for GC-MS measurements of HNC plasma and saliva in the literature that we are aware of, with the next highest being 90.4%^51,52^.

**Table 1:** Top five metabolites identified by variable importance score in the PLS-DA model for cancer vs control in both plasma and saliva.

	Metabolite	Class	Structure
Plasma	Valine	Organic acids and derivatives	
	Histidine	Organic acids and derivatives	
	Tryptophan	Organo-heterocyclic compounds	
	9-Myristoleate	Lipids and lipid-like molecules	
	Malonic acid	Organic acids and derivatives	
Saliva	Trans-4-hydroxyproline	Organic acids and derivatives	
	Propane-1,3-diol	Organic oxygen compounds	
	3-Phosphoglycerate	Organic oxygen compounds	
	1-Monopalmitin	Lipids and lipid-like molecules	
	Azelaic acid	Lipids and lipid-like molecules	

To understand the main metabolic drivers of this diagnostic modeling, we generated similar AUC plots upon systematically removing the metabolites with lowest variable importance score in the PLS-DA model. We found that even using just the top five metabolites for each biofluid achieved AUC scores of 93.0% and 91.2% for plasma and saliva, respectively Supplemental Table S1. The identity and structure of these top five metabolites driving the diagnostic model performance are shown in Table 1.

### Raman spectroscopy analysis

**Figure 2:**
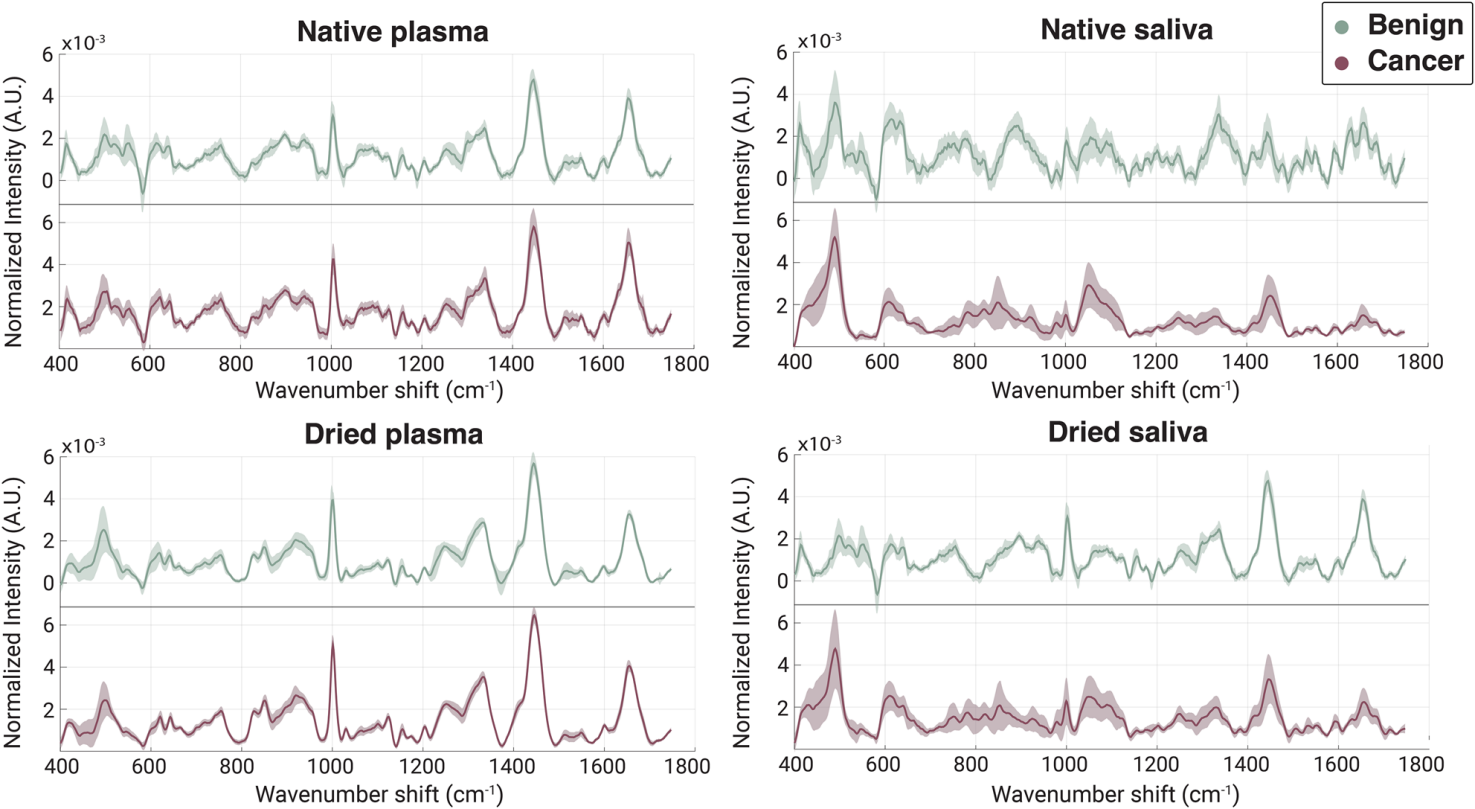
Global averages and standard deviations for cancerous samples (purple) and control samples (green) of the plasma (left) and saliva (right) in both a native (top) and dried (bottom) state. The native samples had a higher degree of standard deviation than the dried samples, indicating there was more heterogeneity across sample measurements.

The full cohort of 53 subject samples was measured using a custom-built inverted Raman confocal microscope. Spectral averages for cancer vs benign control are shown in Fig. 2. Spots were plotted together in a two-dimensional PC space for initial visual assessment. Measurements were taken in both a native (i.e., wet) and dried state, since it is not clear which would be most accurate for downstream diagnostic modeling. Native state measurements are quicker to prepare, yet the weak nature of Raman scattering necessitates a moderately long acquisition time of 30 seconds in unconcentrated samples. During this time, metabolites are diffusing in and out of the focal volume, increasing sampling but also heterogeneity of the signal. On the other hand, drying out the samples under ambient conditions increased sample measurement time by 15 minutes to allow for drying, but concentrates the sample and results in a more stable spectra in the same 30 s. Figure 2 plots the average and standard deviation of the Raman measurements for the two biofluids in both native and dried state.

The dried measurements exhibit less intra- and inter-sample variation compared to the native samples, a trend that is particularly evident for saliva. Comparing dried and native saliva samples, we observed a large discrepancy in the peaks represented by the cancer versus the control group. Raman shifts in the vicinity of 1000 cm^−1^, 1300 cm^−1^, and 1650 cm^−1^ that correspond to key biological materials Table 2 have higher intensity differences between cancer and control in the dried spectra. This indicates that some signals may be more prevalent in the dried state than the native, suggesting that the two methods are probing different chemical aspects of the samples.

**Table 2:** Raman peaks of interest with characteristics in cancer samples (increased or decreased), with assigned functional groups.

Peak/band (cm^−1^)	Cancer	Functional group assignment
492	−	S–S stretch^53^
608	−	CH2 twist^54^
1437	−	CH2 bending in proteins and lipids^55,56^
1526	−	C–N stretching, Amide II^57^
1639	+	Amide I C=O stretching vibrations in proteins^56,32^
488	−	Glycogen^58^
543	+	S–S disulfide bridges in cysteine^57^
679	+	Guanine ring breathing^53^
746	−	C–S aliphatic stretching, Thymine^59^
814	+	Phosphodiester bands^60^
837	−	Amino acids, sugars, and nucleic acids^61^
915	−	Carbohydrate-related SERS vibrations^62^
948	+	C–C, alpha-helix^59^
1000	+	Symmetric ring breathing mode of phenylalanine^60^
1053	−	C–C stretch lipids^58^
1328	+	Amide III-collagen^58^
1445	+	CH2 and CH3 deformations in proteins and lipids^55,56^
1658	+	Amide I (C=O stretching of proteins)/C=C lipid stretch^55,56^
1704	−	Amide I^57^

### Fitting Raman spectra with metabolite standards

Considering that Raman and metabolomics in principle evaluate similar chemical analytes comprising a given sample, we were interested to assess whether the same metabolites driving metabolomics model performance could also be measured in RS. For the ten metabolites shown in Table S2, analytical standards were obtained and measured by RS. Each metabolite was strongly Raman active and showed a distinct spectral signature (representative spectra are shown in Fig. S2).

**Figure 3:**
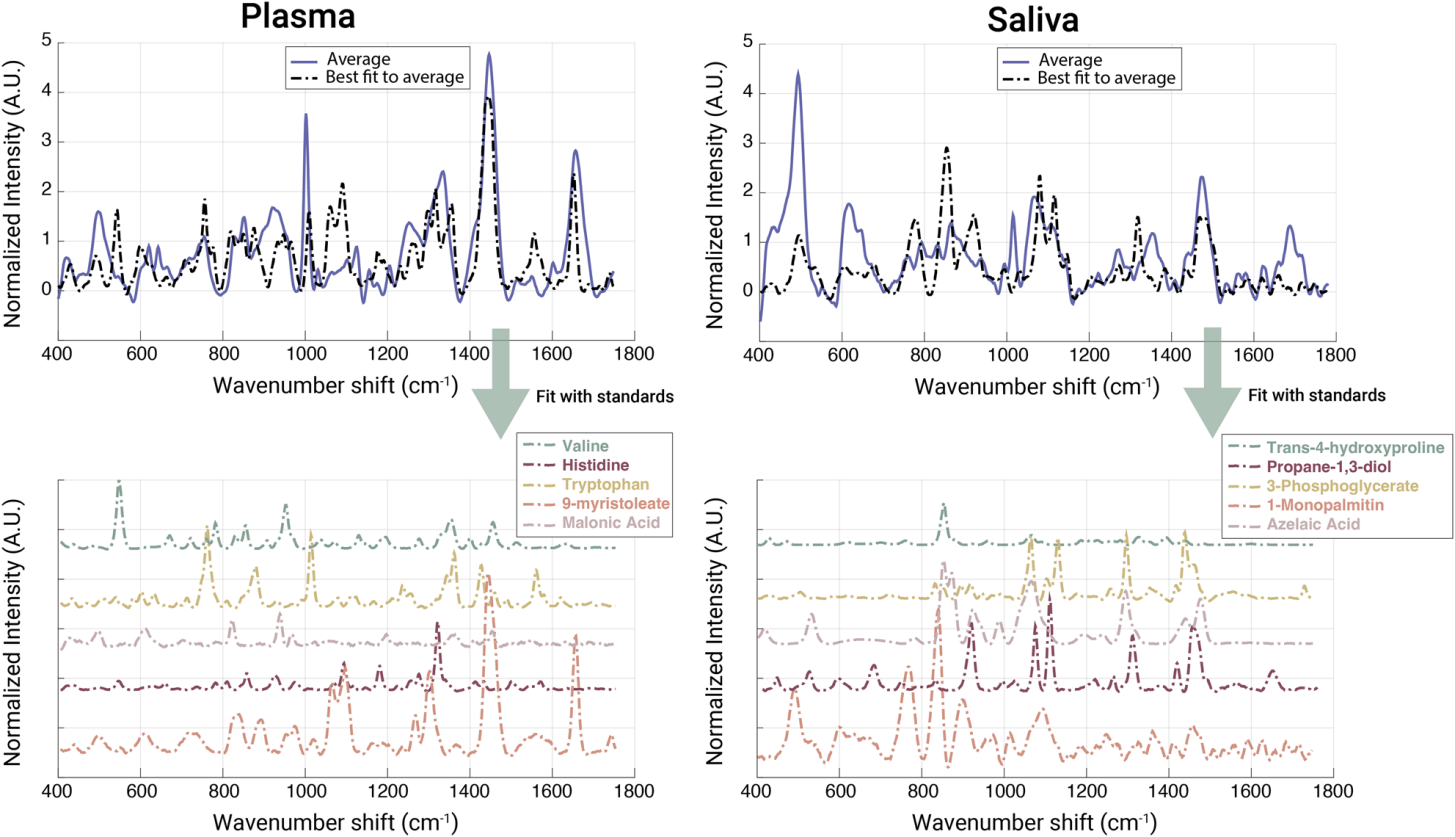
Raman spectra of cancer biofluids contain distinct features that can be attributed to the metabolites identified by GC-MS to drive diagnostic model performance. For each biofluid, average spectra of the cancer samples were fit with their respective metabolites. The average plasma cluster for cancer patients (blue) was fit with the reference spectra below for valine (green), histidine (magenta), tryptophan (yellow), 9-myristoleate (orange), and malonic acid (rose). The average saliva cluster for cancer patients (blue) was fit with the reference spectra below for trans-4-hydroxyproline (green), propane-1,3-diol (magenta), 3-phosphoglycerate (yellow), 1-monopalmitin (orange), and azelaic acid (rose).

To assess their relevance in relation to the Raman data of saliva and plasma, cluster fitting was performed. Principle component analysis (PCA) of all cancer samples for plasma and saliva was carried out. Hierarchical clustering was applied using Euclidean distance metrics considering the top five principal components. The spectra average for the cancer samples was fit (using asymmetric least squares) to combinations of the metabolite standard spectra to assess the extent of which each metabolite contributed to the complex spectra measured for each whole biofluid (Fig. 3). The average spectra for non-cancer samples were also analyzed using the same method but showed a much poorer fit with the metabolite standards, as shown by Supplemental Fig. S3. A significant number of features could be attributed to the metabolites driving the MS model performance, as observed in the Raman average spectra. More specifically, certain spectral features from the metabolite standards had strong fittings with peaks seen in the biofluid spectra. For plasma, the main peaks seen at 1450 cm^−1^ and 1650 cm^−1^ correlate well with the main spectral features of 9-myristoleate. The prominent peak at 1005 cm^−1^ can be attributed to tryptophan. Finally, the spectral feature at 1360 cm^−1^ in valine is also apparent in the cancer spectra. For saliva, the 1450 cm^−1^ shift can be attributed to propane-1,3-diol. The peaks seen at 1045 cm^−1^ and 1091 cm^−1^ fit strongly with features present in azelaic acid, 3-phosphoglycerate, and 1-monopalmitin. Finally, there is good agreement between the peak and the spectral feature seen at 491 cm^−1^ in 1-monopalmitin. Prominent distinct features can be therefore attributed to the various metabolite standards.

In other words, the top five metabolites for each biofluid are more prevalent in the cancer samples than the control samples, indicating that there is good correlation of diagnostic information between the metabolomics and Raman data. It is important to note that the fitting of these metabolites did not account for all spectral variability in either biofluid measurement. For example, in the plasma spectra there was poor fitting at 500 cm^−1^ and 1250 cm^−1^. For saliva, we can see peaks at 615 cm^−1^, 1001 cm^−1^, 1332 cm^−1^, and 1660 cm^−1^ that do not obviously match with the top metabolite standards. While it is apparent that the complex RS spectra contains signatures of the targeted metabolites, much more information is additionally present.

### Diagnostic model performance using Raman spectroscopy

**Figure 4:**
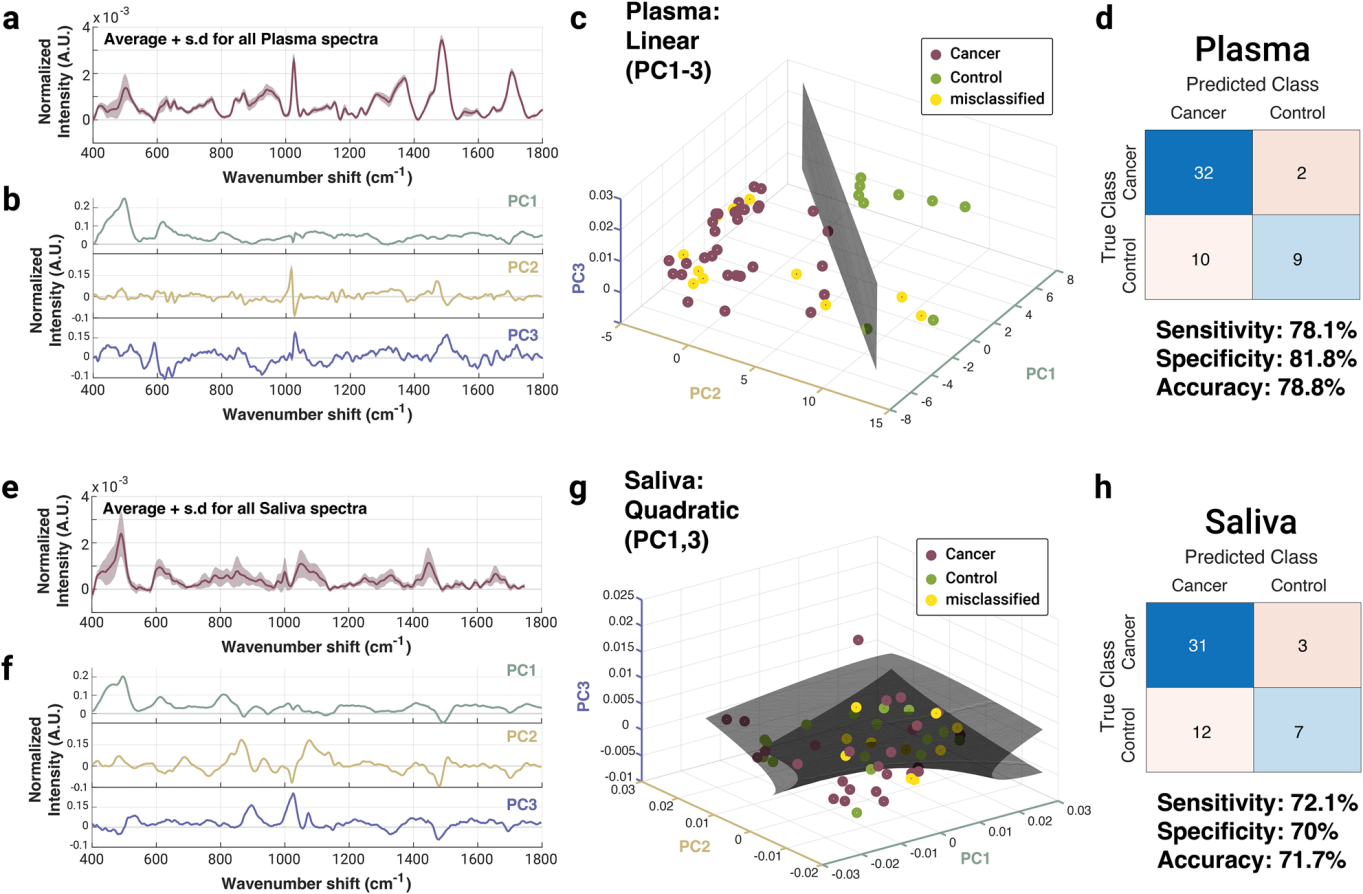
Representative spontaneous Raman data for dried plasma and saliva samples. (**a**) Average spectra and standard deviation for all plasma measurements are plotted. (**b**) Principal component (PC) loadings for PCs 1, 2, and 3 show the major spectral variations amongst the samples. (**c**) The plane of best separation following LDA/QDA algorithm optimization, with cancer (magenta dots) moderately separated from control (green dots) and several misclassified samples (yellow dots). (**d**) 2 × 2 table generated from the best classifier and their associated sensitivity, specificity, and accuracy. (**e**) Average spectra and standard deviation for all saliva measurements are plotted. (**f**) Principal component (PC) loadings for PCs 1, 2, and 3 show the major spectral variations amongst the samples. (**g**) The plane of best separation following LDA/QDA algorithm optimization, with cancer (magenta dots) moderately separated from control (green dots) and several misclassified samples (yellow dots). (**h**) 2 × 2 table generated from the best classifier and their associated sensitivity, specificity, and accuracy.Plasma analysis yielded higher diagnostic capabilities but overall the technical performance was only moderate.

We aimed to assess the ability of spontaneous RS platform to distinguish cancer from control samples in either plasma or saliva samples collected from each subject. Data stacks were generated with all collected spectra (i.e., 5 spots averaged across native and dried saliva or plasma for all 53 study subjects). A representative average plus standard deviation for the dried plasma and saliva samples are shown in Fig. 4a and e respectively. Unsupervised PCA was carried out for each sample typed (e.g., native plasma, dried saliva). Each PC spectrum represents a spectral loading that encompasses a certain amount of the total variation across samples; PC1 has the features responsible for the highest level of variation and each subsequent PC has fewer weight than the previous. The spectral loadings for the first three PCs for the dried plasma and saliva RS measurements are plotted in Fig. 4b and f respectively. Individual patient spectral averages can be re-plotted in PC space (Fig. 4c,g), enabling visualization of supervised data modeling via linear or quadratic discriminant analysis (LDA/QDA). Supervision (i.e., cancer vs benign control) labels were applied based on clinical diagnosis using histopathology analysis. For each sample type, a custom algorithm was run to find the specific flavor of LDA/QDA classifier (using built-in MATLAB classes without hyperparameter optimization) over every combination of the first five PCs (PC1–PC5), which represented 82.5% and 89.7% of the total samples’ variance for plasma and saliva, respectively. For dried plasma, for example, the “linear” discriminant type over PCs 1–3 yielded the highest accuracy, sensitivity, and specificity. The shape of that classifier (reduced to dimensions 1, 2, and 3) is shown in Fig. 4c. Misclassified patients are labeled in yellow. The true (clinician labeled) class vs the RS predicted class in 2 × 2 boxes were used to calculate sensitivity, specificity, and accuracy of the model (Fig. 4d,h). The sensitivities, specificities, and accuracies for dry plasma and saliva were found to be 78.1%, 81.8%, and 78.8%, and 72.1%, 70%, and 71.7% respectively. For native saliva and plasma, the sensitivities, specificities, and accuracies were 82.8%, 58.3%, and 71.7%, and 78.6%, 90.1%, and 81.1% respectively. More detailed information about the groups and the classifiers used for each can be found in Supplementary Table S3.

**Figure 5:**
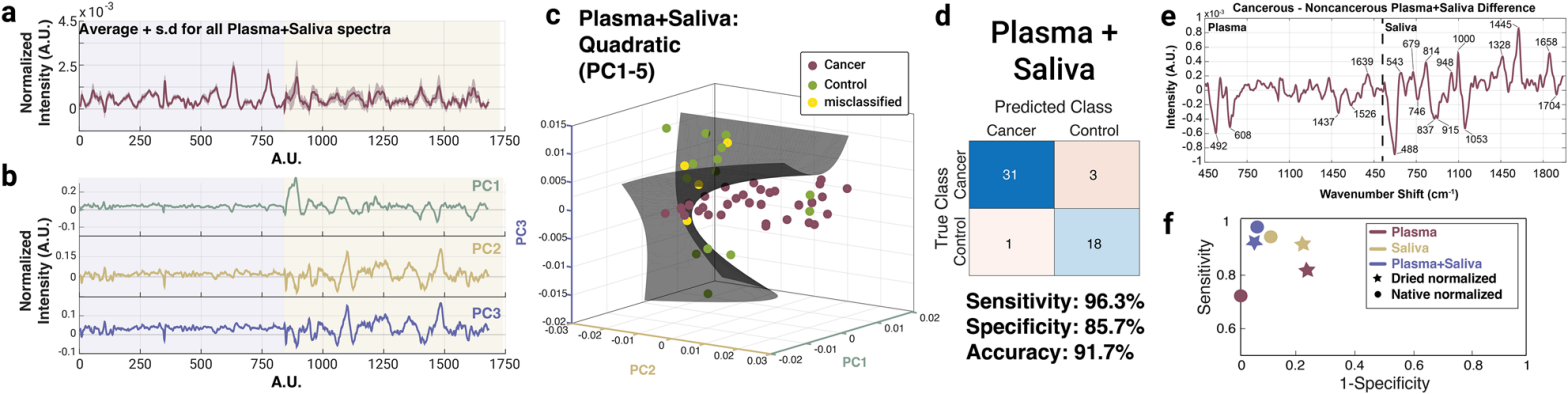
Representative spontaneous Raman data for combined plasma and saliva dried samples. (**a**) Global average and standard deviation of the plasma and saliva combined spectra. The blue overlay represents the portion of the spectra that is plasma, and the gold overlay represents the portion that is saliva. (**b**) Principal component (PC) loadings for the first three PCs showing the different areas of variation between the plotted samples. (**c**) 2 × 2 table created using the Quadratic PC1–5 classifier to assess sensitivity, specificity, and accuracy, of which were 96.3%, 85.7%, and 91.7% in this group. (**d**) Spectrum representing the peaks obtained from subtracting the average control spectrum from the average cancer spectrum for combined plasma and saliva. Important spectral features were identified and labelled. (**e**) Plot of all combinations (native and dried, combined and individual) sensitives vs. 1-specificity. The combined biofluids outperformed the single biofluids for most of the categories.

Although plasma outperformed saliva in separating cancer from non-cancer, both biofluid groupings had lower diagnostic capability than the metabolomics results showed. We were then curious to see if simple data augmentation methods could improve performance. Therefore, we stitched average spectra across both biofluids together for each patient to create a single unified spectrum. The intensity values from the saliva samples were copied and added to the backend of the plasma samples to create these new combined biofluid spectra. X-axis values were reassigned as arbitrary numbers from 1 to 1694 (the horizontal CCD pixels doubled after merging the data together). For each of the 53-subject cohort (34 HNC cancer patients and 19 benign controls), the same procedure of averaging each spot to produce one spectrum per patient was followed and the samples were once again projected into the PC space to create a stack with the PC1-5 associated values. The global averages and standard deviations of this new group for all dried samples is shown in Fig. 5a with the first three PC loadings shown in (Fig. 5b). With the best classifier (quadratic with PC 1–5), we achieved a remarkable sensitivity, specificity, and accuracy of 96.3%, 85.7%, and 91.7% (Fig. 5c). The sensitivity vs. 1-specificity for all groups (both native and dried, single biofluid and combined biofluid) are presented in Fig. 5e. As evidenced by this graph, the saliva alone tends to have better performance than the plasma when analyzed individually, but the performance of the plasma and saliva combined is superior to the individual biofluid groups. These combined biofluids give diagnostic ability on par with the metabolomics results, supporting our Raman system, combined with simple data augmentation, as a strong diagnostic approach.

Identification of the spectral features driving model performance is a powerful way to glean deeper chemical information from samples. To investigate what chemical entities were the highest drivers of spectral importance, we created a representative composite spectrum of the combined plasma and saliva specimens by averaging all the cancer and control samples and then calculating the difference. The resulting spectrum is shown in Fig. 5d with the most influential peaks labelled. The saliva section of the combined spectrum contained far more meaningful peaks than the plasma section. We further analyzed the chemical signatures associated with each peak using the tentative assignments shown in Table 2. Although efforts to assign the vibrational peaks prominent in the spectrum have been made, we caution against overinterpretation of single spectral peaks. Instead, we consider the significance of major groupings of distinct feature types such as carbohydrates, proteins, lipids, or nucleic acids.

The majority of the peaks identified in the plasma portion (e.g. 492 cm^−1^, 608 cm^−1^, 1437 cm^−1^, and 1526 cm^−1^) correspond to protein vibrational modes. Further, all besides one are negative which indicates these protein signatures are more commonly seen in the non-cancerous samples than the cancerous ones. This indicates that important distinct differences between the two sample types are reflected in protein composition. This notion is further supported when reflecting back to the top five metabolites identified for plasma from the GC-MS measurements. Of the five, three are amino acids, the building blocks of proteins and only one comes from a class associated with lipids, strengthening the idea that protein content is generally driving the differences between the cancer and control samples. We also identified the peaks that can be attributed to the metabolites themselves. For example, the best fit to the average line shown in Fig. 3 (which contains all five metabolites together) reflects key protein peaks at 492 cm^−1^, 1437 cm^−1^, and 1639 cm^−1^). Two distinct peaks from tryptophan (877 cm^−1^^56^, and 1556 cm^−1^^55^) are present in the fitting spectra but not in the plasma section of the stitched data. There were also peaks at 602 cm^−1^^32^, 1060 cm^−1^^58^, 1090 cm^−1^^55^, and 1179 cm^−1^^48^ that are contributing to C–C stretches in lipids and proteins. These absence of these peaks in Fig. 5d indicates they did not play a strong diagnostic role after the biofluid stitching procedure was complete.

In the saliva section of the composite spectrum, we also observed notable chemical groupings. Strong signals at 543 cm^−1^ (S–S disulfide bridges in cysteine), 679 cm^−1^ (guanine ring breathing), 1000 cm^−1^ (symmetric ring breathing in phenylalanine) and 1445 cm^−1^ (CH2 and CH3 deformations in proteins) are seen with positive values, once again aligned with the idea that the protein profile of cancer and control samples differ widely. Peaks are 746 cm^−1^ (C–S aliphatic stretching) and 1053 cm^−1^ (C–C stretch in lipids) speak to the general lipid profile and are negative, indicating their commonality in the non-cancerous spectra. Taken together the positive protein and negative lipid peaks may demonstrate the necessity to analyze general ratios of these components present in future samples. Once again there were analogous peaks between those identified above and the average fitting spectra from the saliva metabolites shown in Figure 3, including 543 cm^−1^, 837 cm^−1^, 1053 cm^−1^, and 1445 cm^−1^. However, we similarly observe a rise of peaks specific to the fitting spectra that did not appear in the stitched data. These were 774 cm^−1^ (nucleic acids)^55^ , 845 cm^−1^ (polysaccharides)^55^, 908 cm^−1^ (skeletal C–C in lipids)^54^, 1100 cm^−1^ (C–C stretch in lipids)^57^, and 1296 cm^−1^ (fatty acids)^55^. These results indicate that specific features of the metabolites are helping identify the cancerous samples more directly than others, and serve as the potential chemical drivers contained within the diagnostic information.

Perhaps the most interesting thing to note is the exceptionally negative peak at 488 cm^−1^. This peak is associated with glycogen, a polysaccharide that serves as a main form of energy storage. The negative value here argues that this peak is very prominent in non-cancerous samples but not cancerous ones. This is interesting to see considering cancer cells undergo aerobic glycolysis (referred to as the Warburg effect) to promote rapid and continuous growth^63^. Human Papilloma Virus (HPV) is a large mediator of new HNC cancers, and there are many associated HPV proteins that activate specific proteins or pathways within the body to assist in the switch to aerobic glycolysis^63^. These include epidermal growth factor receptor (EGFR), protein complex mTORC2, and the retinoblastoma protein (Rb)^63^. Interestingly, it has been shown that when HPV viral proteins interact with these groups, the level of glycogen present drastically decreases^63^. This in combination with the fact that the glycogen peak presents as negative in the composite spectrum support the notion that glycogen signal could be a strong diagnostic indicator.

We dug deeper to examine the particular patients who were being misclassified within all the different groups and classifiers for the original cohort of 28 patients. Table S4 outlines the patients tested as well as where they were misclassified. It is apparent from this table that although there is some patient overlap, most groups seem to be misclassifying different patients—there was no obvious parity. Further, more plasma samples were misclassified than saliva samples, although very few were misclassified with the combined biofluid groups (as evident by the model performance). Perhaps not surprising is also the trend that native conditions had more misclassifications than the dried conditions did. This could again be attributed to the inherently higher heterogeneous signal obtained in the native state. Since the particles and entities in the biofluid are moving in and out of the focal volume during measurements, it is more difficulty to get a consistent snapshot of the chemical signal of the sample. Others attempting to utilize our methods for diagnostic application of RS may find it beneficial to examined dried samples as compared to native.

Within the cohort of cancer patient samples, we attempted to carry out discrimination of cancer staging, i.e., split into two groups of early (stage I/II) vs late (stage III/IV), as assessed by the clinician. But the performance of our models was poor. In future work with a higher number of samples in each category, we will apply more sophisticated data models to try to elucidate stage-based discrimination. While we did not perform SERS in this study, this may be a future area of interest where stitching data across biofluids could improve performance metrics.

## Discussion

The accuracy of HNC diagnostics using Raman spectroscopy is highlighted in several recent works. In direct analysis of whole tissues, Jeng et al. reported an accuracy of 81.25%, sensitivity of 77.27%, and specificity of 86.11% for discrimination of cancerous versus healthy samples on a cohort of 80 total patients^64^. Yan et al. was able to increase these numbers to the high 90s through implementation of a machine learning algorithm, albeit on a small dataset of only 12 patient samples^26^. A systematic review of using Raman for oral cancer diagnostics by Zhan, et al also describes a meta-analysis of 41 articles, citing that the accuracy of RS in oral cancer diagnostics on in vitro frozen tissues as 99.68%^25^. Although these are impressive numbers, the clinical relevance for diagnostics is lacking, as these studies do not address the need to create fast, non-invasive liquid biopsy approaches for rapid diagnostics. Another group performed RS measurements on HNC saliva samples from a cohort of 32 patients with accuracy of 90%, which was lower than the numbers reported from the groups analyzing tissue but still clinically useful^24^. Our stitched biofluid accuracy, sensitivity, and specificity slightly outperforms that study across a much large patient cohort.

Recent reviews have investigated the application of metabolomics to HNC diagnostics and provide a thoughtful summary of where the field stands^65,66^. Metabolomics analysis of HNC has been carried out in many different biofluids ranging between urine^67^ to serum^68–71^ to saliva^72–75^. Saliva as a biofluid source is of interest for many reasons: first, it is non-invasive and easily obtained from patients; second, low volumes are needed for metabolomic analysis, and third, the close proximity saliva has to the HNC tumors may provide additional biomarker information and contain a higher level of cancer metabolites of interest. Although a few studies have been carried out that show saliva contains important features that can distinguish cancer from non-cancer, there is little agreement of which metabolites are important when comparing the results across studies. Furthermore, the dynamic nature of saliva and contamination from recent diet may pose issues for accurate detection of tumor biomarkers. Few studies have been carried out using RS directly on saliva from HNC samples^24,76,77^. The studies that have been conducted are diverse in their methodologies and results, making it hard to provide concrete evidence that RS analysis of saliva is a viable diagnostic tool. Further, there are many variables that influence the profile of saliva collected from patients, including age^78^, smoking habits^79^, time of day^80,81^, fasting regimen^81^, and gender^78^. Controlling for all of these factors is necessary for clinical adaptation.

Bringing RS into the clinic is attractive for many reasons. RS can be performed in aqueous solutions without the interference of a water signal that can hinder other spectroscopic techniques such as infrared spectroscopy. Collection and separation of biofluids from patients undergoing treatment is common and being able to perform point-of-care measurements directly on those biofluids in near real time creates an obvious advantage for RS systems. Metabolomics remains the gold standard for biomarker discovery due to its ability to develop rich chemical data libraries^5^. However, there are drawbacks to using this technology as the main tool for such investigation. Perhaps the most vital drawback is the challenge of validating the identified metabolites robustly across many datasets. The analysis of samples and production of results may be relatively straightforward, but the potential biomarkers cannot be used unless they are rigorously validated to report on the disease state.

By combining each patient’s Raman data from their plasma and saliva samples into a single spectrum, we dramatically improved the diagnostic value of spontaneous RS, a key finding of this study. These data indicate that future work should focus on the development of methods that can provide a more comprehensive view of the physiological state of an individual’s health. Further, we showed that the same metabolites identified as driving diagnostic model performance in GC-MS could be correlated by RS performed in the very same samples, providing a level of validation that many other technologies lack. Our findings validate that RS is inherently measuring a similar subset of metabolites compared to GC-MS, confirming them as promising biomarkers for HNC. Yet is also establishes that the spectral features themselves, divorced of assignment to any particular metabolite species (and their concentrations), are highly capable of driving accurate diagnostics. Although RS, even for the combined biofluid data augmentation, performed slightly worse in terms of accuracy compared to GC-MS, RS is comparatively easier, quicker, uses less sample volume, is non-destructive, requires minimal sample prep, and is less expensive to carry out.

## Conclusions

In this study, we analyzed a robust clinical dataset of 53 HNC patient and benign control liquid biopsy samples to classify disease. Metabolomics measurements were performed using GC-MS as a gold standard of comparison. GC-MS results yielded a 95.9% (plasma) and 92.4% (saliva) cancer vs. control discrimination using PLS-DA. The top five metabolites identified for each biofluid were further studied using our custom Raman scope. We found that the prevalence of these metabolites was higher in all cancer samples versus the control, validating that RS is inherently sensitive to the same metabolites driving excellent GC-MS model performance. We further tested the ability of our Raman platform to separate cancer from control within the individual biofluids. With a pseudoquadratic classifier we achieved sensitivities and specificities in the 70–90% range. Instead, with simple data augmentation to stitch plasma and saliva datasets together to create a single, integrated spectrum, sensitivity, specificity, and accuracy increased to 96.3%, 85.7%, and 91.7%. The results of this study indicate an exciting step in validating Raman spectroscopy as a robust diagnostic tool, as well as introducing a new finding that a holistic view of an individual’s sample (in this case, a combination of their plasma and saliva) can provide a greater level of information indicative of specific disease states.

## Supplementary Information

Supplementary Information.

## Data Availability

All raw datasets generated and analyzed during the current study are freely available on a Zenodo repository with the identifier 10.5281/zenodo.7044324. MATLAB code used to process the datasets are available from the corresponding author on request.
