# Supplementary material for: Fused Raman spectroscopic analysis of blood and saliva delivers high accuracy for head and neck cancer diagnostics
Source: Sci Rep. 2022 Nov 2;12:18464. doi: 10.1038/s41598-022-22197-x (PMC9630497; doi:10.1038/s41598-022-22197-x)
Supplement: Supplementary file 1 — Supplementary Information. [file 41598_2022_22197_MOESM1_ESM.pdf]

Electronic Supplementary Material

Table 1 Clinical biofluid information with demographics data. An ‘X’ indicates whether plasma or saliva was collected for each patient.

Patient ID	Plasma	Saliva	Diagnosis	Gender	Age
OD-0272	X	X	Cancer	Male	73
OD-0275	X	X	Cancer	Male	86
OD-0276	X	X	Cancer	Male	63
OD-0277	X	X	Non-cancer	Male	30
OD-0278	X	X	Non-cancer	Male	27
OD-0279	X	X	Cancer	Male	28
OD-0280	X	X	Cancer	Male	66
OD-0281	X	X	Non-cancer	Female	43
OD-0284	X	X	Cancer	Male	53
OD-0286	X	X	Non-cancer	Male	63
OD-0287	X	X	Cancer	Male	72
OD-0288	X	X	Non-cancer	Female	31
OD-0289	X	X	Cancer	Female	74
OD-0291	X	X	Non-cancer	Male	72
OD-0292	X	X	Cancer	Male	51
OD-0293	X	X	Non-cancer	Male	50
OD-0294	X	X	Non-cancer	Female	44
OD-0295	X	X	Cancer	Female	71
OD-0296	X	X	Cancer	Male	66
OD-0297	X	X	Cancer	Male	77
OD-0298	X	X	Cancer	Male	81
OD-0300	X	X	Cancer	Male	79
OD-0301	X	X	Cancer	Male	57
OD-0302	X	X	Non-cancer	Female	70
OD-0304	X	X	Cancer	Male	67
OD-0305	X	X	Cancer	Male	58
OD-0306	X	X	Cancer	Male	56
OD-0310	X	X	Cancer	Male	65
OD-0312	X	X	Cancer	Male	63
OD-0313	X	X	Cancer	Male	64
OD-0314	X	X	Cancer	Male	57
OD-0315	X	X	Cancer	Female	72
OD-0316	X	X	Cancer	Male	65
OD-0318	X	X	Cancer	Female	78
OD-0319	X	X	Cancer	Female	62
OD-0320	X	X	Cancer	Male	54
OD-0321	X	X	Non-cancer	Female	47
OD-0322	X	X	Cancer	Male	69
OD-0324	X	X	Cancer	Male	57
OD-0326	X	X	Non-cancer	Female	58
OD-0327	X	X	Cancer	Male	61
OD-0328	X	X	Cancer	Female	71
OD-0329	X	X	Cancer	Female	72
OD-0330	X	X	Cancer	Female	30
OD-0331	X	X	Cancer	Male	76
OD-0334	X	X	Cancer	Male	67
OD-0335	X	X	Cancer	Female	76
OD-0336	X	X	Non-cancer	Female	66
OD-0337	X	X	Non-cancer	Female	58
OD-0339	X	X	Non-cancer	Male	65
OD-0340	X	X	Non-cancer	Male	29
OD-0341	X	X	Non-cancer	Female	57
OD-0342	X	X	Non-cancer	Male	37
OD-0345	X	X	Non-cancer	Male	34

Table 2 The biofluid group along with the best identified classifier, and the accompanying accuracy, sensitivity, and specificity achieved with it.

Group	Best Classifier	Accuracy, Sensitivity, and Specificity
Dry Plasma	1,2,3,5 Diaglinear	79.3%, 78.3%, 83.3%
Native Plasma	1,2,3,4,5 Quadratic	81.4%, 79.1%, 90.1%
Dry Saliva	2,5 Diagquadratic	71.7%, 72.1%, 70%
Native Saliva	1,3,4 Quadratic	71.7%, 82.8%, 58.3%
Dry Combined	1,3,4,5 Quadratic	86.3%, 90.3%, 80%
Native Combined	1,2,3,4,5 Quadratic	91.7%, 96.3%, 85.7%

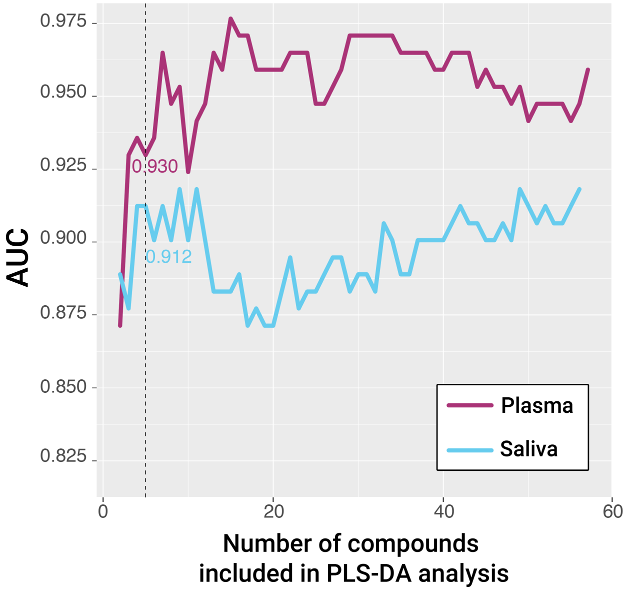

Fig. 1 Area under curve (AUC) values with increasing number of metabolites as detected by GC-TOF-MS included in the PLS-DA model according to variable importance score for plasma (purple line) and saliva (blue line). The dotted black line indicates the AUC values for the PLS-DA model incorporating the top 5 metabolites for each biofluid, with their corresponding AUC values labeled.

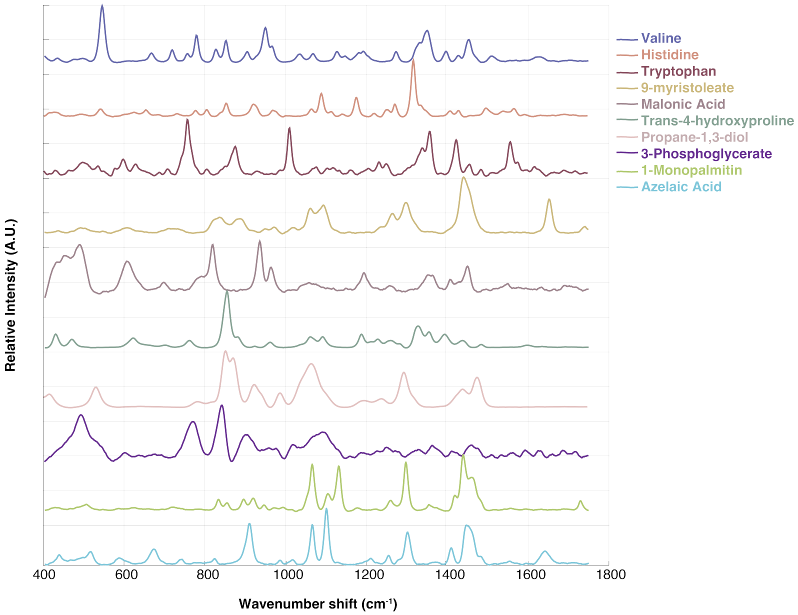

Fig. 2 Raman spectra of the ten metabolites identified from GS-TOF-MS measurements grouped by their identification in PLS-DA models from either plasma (top five) or saliva (bottom five). Within each group, they are listed in order from most prevalent to least with respect to their variable importance score in the PLS-DA model. Valine, histidine, tryptophan, 9-myristoleate, and malonic acid were identified from plasma and trans-4-hydroxyproline, propane-1,3-diol, 3-phsophoglycerate, 1-monopalmitin, and azelaic acid were identified from saliva.

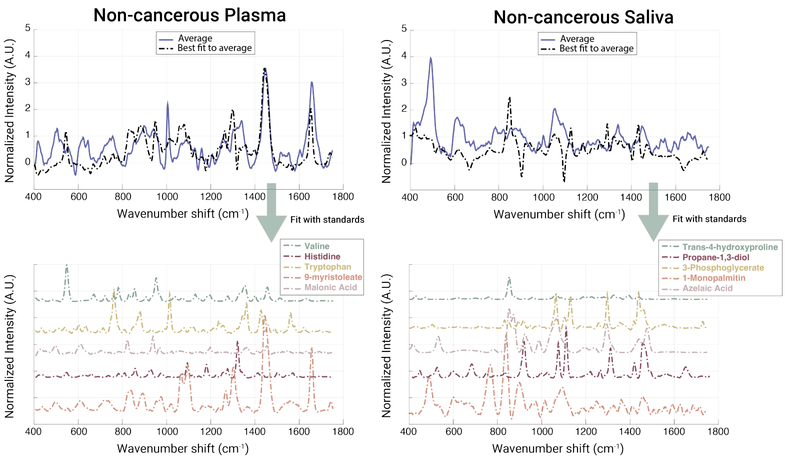

Fig. 3 Raman spectra of non-cancer biofluids contain fewer features that can be attributed to the metabolites identified by GC-MS to drive diagnostic model performance. For each biofluid, average spectra of the non-cancer samples were fit with their respective metabolites. The average plasma cluster for non-cancer patients (blue) was fit with the reference spectra below for valine (green), histidine (magenta), tryptophan (yellow), 9-myristoleate (orange), and malonic acid (rose). The average saliva cluster for non-cancer patients (blue) was fit with the reference spectra below for trans-4-hydroxyproline (green), propane-1,3-diol (magenta), 3-phosphoglycerate (yellow), 1-monopalmitin (orange), and azelaic acid (rose).

Table 3 Individual patients and which groups they were misclassified in.

Sample	GC-MS plasma	GC-MS Saliva	Dried plasma	Native plasma	Dried saliva	Native saliva	Dried combined	Native combined
OD-0272						X		
OD-0275								
OD-0276						X		
OD-0277				X	X			
OD-0278		X	X	X	X		X	
OD-0279								
OD-0280					X			
OD-0281								
OD-0284								
OD-0286			X	X	X			
OD-0287								
OD-0288			X	X				X
OD-0289								X
OD-0291			X	X			X	
OD-0292								
OD-0293				X	X	X		
OD-0294			X	X	X			
OD-0295					X			
OD-0296			X					
OD-0297			X	X			X	X
OD-0298						X		
OD-0300					X			
OD-0301	X							
OD-0302			X		X			
OD-0304					X			
OD-0305						X		
OD-0306						X		
OD-0310								
OD-0312								
OD-0313						X		
OD-0314								
OD-0315								
OD-0316						X		
OD-0318						X		X
OD-0319								
OD-0320								
OD-0322						X		
OD-0324							X	X
OD-0325								
OD-0326			X		X	X		
OD-0327								
OD-0328								
OD-0329						X	X	
OD-0330							X	
OD-0331			X	X	X	X		
OD-0334								
OD-0336					X			
OD-0337					X	X		
OD-0339								
OD-0340					X			
OD-0341								
OD-0342					X	X		
OD-0345								
